# Rapid Reviews: A Tutorial

**DOI:** 10.1002/cesm.70092

**Published:** 2026-07-17

**Authors:** Chantelle Garritty, Candyce Hamel, Barbara Nussbaumer‐Streit

**Affiliations:** ^1^ School of Epidemiology and Public Health University of Ottawa Ottawa Ontario Canada; ^2^ Global Health and Guidelines Division Public Health Agency of Canada Ottawa Ontario Canada; ^3^ Ottawa Hospital Research Institute Methodological and Implementation Research Ottawa Ontario Canada; ^4^ Cochrane Austria, Department for Evidence‐based Medicine and Evaluation University for Continuing Education Krems Krems an der Donau Austria

## Abstract

This tutorial provides an overview of the steps involved in conducting rapid reviews of interventions based on recent methodological guidance and empirical studies from the Cochrane Rapid Reviews Methods Group (RRMG). This tutorial provides practical guidance on methods, key decisions, and strategies to optimize the balance between timeliness and rigor and is intended as a practical introduction for researchers, clinicians, and policymakers interested in conducting or using rapid reviews.

## Graphical abstract

This tutorial provides an overview of the steps involved in conducting rapid reviews of interventions based on recent methodological guidance and empirical studies from the Cochrane Rapid Reviews Methods Group (RRMG). This tutorial provides practical guidance on methods, key decisions, and strategies to optimize the balance between timeliness and rigour and is intended as a practical introduction for researchers, clinicians, and policymakers interested in conducting or using rapid reviews.

https://links.cochrane.org/cesm/tutorials/rapid-reviews

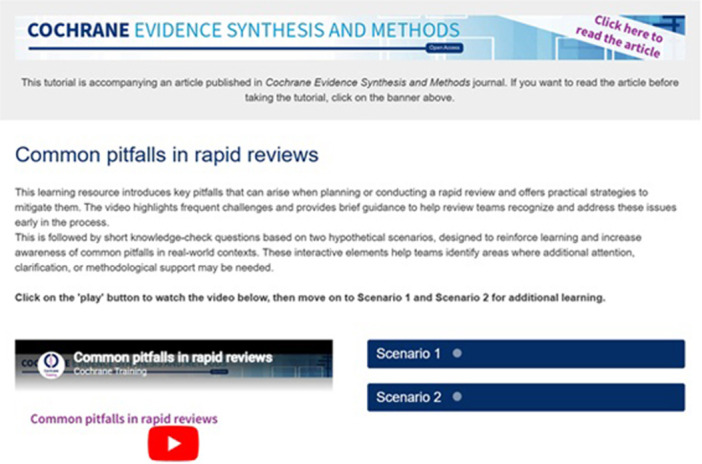

## 1 What Is a Rapid Review?

A rapid review is a streamlined evidence synthesis produced faster than full systematic reviews by omitting or abbreviating specific processes. Therefore, rapid reviews aim to provide timely and relevant evidence synthesis while maintaining the highest possible level of rigor within existing constraints, by streamlining one or more review steps [1]. Their utility has grown significantly in response to urgent health crises, time‐sensitive policy decisions, and resource‐constrained environments. While speed and abbreviated methods are defining features, maintaining methodological integrity and transparency remains critical. Well‐established Cochrane rapid review methodological guidance exists and serves as a strong, evidence‐informed reference framework for conducting rapid reviews of interventions [1]. This guidance draws on both empirical methodological research and expert consensus in areas where evidence is limited.

Typically completed within weeks to a few months (up to 6 months per Cochrane rapid reviews methods guidance) [1], rapid reviews:

- Are driven by urgent healthcare decision needs and are often initiated by interest holders
- Restrict scope (interventions, outcomes, study types)
- Use abbreviated processes (e.g., searching fewer databases, single reviewer screening)

**Key distinct features compared to full systematic reviews include:**

1. Planned methodological abbreviations
2. Timeline‐sensitive conduct

## 2 Deciding If a Rapid Review Is Appropriate

Rapid reviews are not suitable for every context. Before embarking on a rapid review, it is essential to assess whether the approach is appropriate. General criteria are available to help determine when a rapid review is appropriate in Table 1 [2]. Rapid reviews are best suited for focused questions with limited scope, often requiring urgent results (i.e., within a few weeks up to 6 months). Otherwise, a full systematic review is suggested.

**TABLE 1:** General criteria for determining when a rapid review is appropriate.

Criterion	Description
Urgency	Decision required within weeks to months (e.g., emerging health crisis)
Scope and question focus	Specific and limited scope that allows for rapid completion while still answering the question being asked, and a well‐defined PICO (Population, Intervention, Comparator, Outcome)
Available resources and expertise	Skilled team (i.e., expertise in systematic review methods, clinical or subject matter knowledge, and information specialist), software tools, access to databases, and the technology and resources to complete the review within the required timeframe
Interest holder alignment of trade‐offs	Interest holders understand and accept methodological abbreviations and their potential biases

## 3 Core Steps in a Rapid Review

### 3.1 Step 1: Engaging Interest Holders and Defining the Question

Often initiated by interest holders, it is important to engage them, such as patients, caregivers, the public, healthcare providers, and policymakers, to help prioritize outcomes, refine eligibility criteria, and determine timelines [3].

- Use the PICO framework (Population, Intervention, Comparator, Outcome), which is suitable for rapid reviews of intervention questions.
- Limit the number of outcomes (≤ 7 primary outcomes recommended, including at least one outcome for effectiveness and one for harms)
- Predefine subgroups or context‐specific considerations

Interest holders should be consulted early and at key points to ensure relevance. While engagement may be challenging within rapid review timelines, teams should make reasonable efforts to include at least some representative interest holders (including patients, where appropriate). Involving interest holders may add time, but this can be mitigated through early planning and by maintaining a small group of participants available for rapid input.

### 3.2 Step 2: Protocol Development

Develop and, ideally, register a protocol to ensure transparency and team coordination. While some flexibility is necessary due to iterative timelines, protocols should document [4]:

- Review question
- Eligibility criteria (e.g., study design, population, interventions, control, outcomes)
- Search strategy overview
- Planned methods of conduct (e.g., data extraction)
- Deviations from full systematic review methodology

Protocol registration (e.g., PROSPERO, Open Science Framework) is encouraged but may be adapted to suit condensed timelines.

### 3.3 Step 3: Literature Searching

Create an effective search that balances speed and comprehensiveness. The Cochrane RRMG recommends [5]:

- Searching at least two core databases (e.g., MEDLINE, CENTRAL, Embase)
- Engaging an information specialist (e.g., medical librarian) to develop tailored search strategies
- Using pre‐tested search strings from existing systematic reviews, if available
- Applying filters for date, language, or study design with clear justification
- Perform supplementary searches (e.g., citation tracking, gray literature), dependent on topic urgency and resource availability.

To ensure high‐quality and transparent search strategies in rapid reviews:

- Use the PRESS (Peer Review of Electronic Search Strategies) guideline [6], where an experienced information specialist peer reviews the search strategy to help identify errors and improve comprehensiveness before it is finalized.
- Follow PRISMA‐S to report the search process clearly and completely, including databases searched, limits used, and provide full strategies [7].

### 3.4 Step 4: Study Selection

The following are recommended [4]:

- Pilot the screening process (e.g., 20–50 citations) to ensure consistency.
- Dual screening of a proportion of records (e.g., 20%) and if agreement among reviewers is considered sufficiently high (e.g., based on Cohen's kappa ≥ 0.80), move on with single screening. However, kappa statistics also have recognized limitations and should be interpreted in the context of the review process, prevalence of included studies, and complexity of the eligibility criteria.
- Dual screening when feasible due to limited number of retrieved records (titles/abstracts and full texts).
- Maintain detailed records of inclusion/exclusion decisions (PRISMA flow chart).

### 3.5 Step 5: Data Extraction

Focus on pre‐specified, decision‐relevant outcomes. The following are recommended [4]:

- Use standardized and piloted forms.
- One reviewer extracting data with verification by a second reviewer.
- Extract only essential data related to the primary outcomes (population details, intervention characteristics, and key results).

Data from existing systematic reviews may also be used in rapid reviews when they are directly relevant to the question and meet eligibility criteria, have transparent and rigorous methods (i.e., not critically flawed) as assessed using validated appraisal tools (e.g., AMSTAR 2, ROBIS), and are sufficiently current for the decision context. Whether a review is considered sufficiently current will depend on the topic and decision‐making context, as some research areas evolve more quickly and may become outdated sooner, whereas evidence in other fields may remain applicable and relevant for longer periods. For systematic reviews, the date of the last literature search is a more important indicator of currency than the publication date itself when determining whether the evidence remains sufficiently up to date for the review question and context.

### 3.6 Step 6: Risk of Bias Assessment

Assess risk of bias, which remains essential in rapid reviews despite the time constraints [4]. Recommended validated tools include, for example:

- RoB 1.0 or RoB 2.0 for randomized controlled trials [8, 9]
- ROBINS‐I for non‐randomized studies [10]

Single‐reviewer assessment with verification by a second reviewer is acceptable.

### 3.7 Step 7: Synthesis and Analysis

Synthesize the results narratively, at a minimum. Meta‐analysis may be conducted when data are sufficiently homogenous and time permits. Key principles to follow are [11]:

- Group studies by intervention or outcome
- Use summary tables and harvest plots to support narrative interpretation
- Discuss heterogeneity and consistency

Cochrane's RRMG emphasizes the use of summary of findings tables and plain‐language summaries to facilitate interpretation by end‐users.

### 3.8 Step 8: Certainty of the Evidence

Assess the certainty of the evidence, which helps readers understand not only what the evidence shows but also how confident they can be in those findings. The GRADE (Grading of Recommendations, Assessment, Development and Evaluation) framework is the preferred structured and transparent method for this purpose. In a rapid review context, review teams may [12]:

- Limit GRADE to key outcomes and interventions
- Have one person complete the GRADE assessment, with a second person to verify the assessment

If applying the GRADE approach is not possible, reviewers should state that clearly and still provide comments on the overall confidence in the evidence.

### 3.9 Step 9: Use of Digital Tools

Improve efficiency and reproducibility with the use of digital tools, including automation tools [13]. Table 2 shows examples of useful platforms and tools. The list is meant as a guide and is not exhaustive.

**TABLE 2:** Useful tools in rapid reviews.

Steps	Tools	Description
Step 3. Documenting search results	EndNote, Zotero	Reference management (to collect, organize, cite and share bibliographic information)
Step 4. Study selection	Covidence, DistillerSR, EPPI‐Reviewer, Rayyan, Sustained Knowledge Platform (SKP) by Epistemonikos	Title/abstract and full‐text review
Step 5. Data extraction	Covidence, DistillerSR, EPPI‐Reviewer, Excel, Google Sheets	Customizable forms and audit trails (in some software)
Step 7. Certainty	GRADEpro	GRADE evidence profiles
Step 8. Synthesis	RevMan, Meta‐Essentials, EPPI‐Reviewer	Meta‐analysis and evidence mapping
Real‐time tools/Living documents	Zoom, Slack, Google Docs, Teams	Collaborative platforms for team communications and real‐time co‐authoring centralizing feedback

Machine learning classifiers and automation tools (e.g., Covidence, DistillerSR) can assist in prioritizing records but must be validated against human judgment. Moreover, large language models (LLMs), such as ChatGPT or Gemini, may be applied to support specific steps in the rapid review process (e.g. drafting text or screening assistance). However, the RRMG advises that their use must be approached with responsibility and caution. This is consistent with the position statement of the Cochrane Rapid Reviews Methods Group (RRMG) [14] and with Cochrane's evolving RAISE guidance, which emphasizes *Responsible AI use in Systematic Evidence synthesis* [15], ensuring transparency, human oversight, and careful consideration of potential risks and biases when integrating AI tools.

### 3.10 Step 10: Reporting and Dissemination

Transparent reporting is essential to ensure that findings can be accurately interpreted and replicated. To support this, the RRMG has established interim guidance for the reporting of rapid reviews [16]. In addition, the RRMG recommends using the following established reporting guidelines:

- PRISMA‐P for protocol development [17]
- PRISMA‐S for documenting the search strategy [7]
- PRISMA 2020 for reporting the conduct and results of the review [18]

While these tools were originally developed for full systematic reviews, they may be used and should be adapted as appropriate for use in rapid reviews.

All deviations from standard methods should be clearly explained. In addition, the rationale for doing the rapid review and the timeline to conduct the review should be reported. A table summarizing deviations from standard methods may be helpful [16].

In addition to a peer‐reviewed publication, authors may prepare:

- One‐page summaries for policymakers
- Slide decks or infographics targeted to specific interest holder audiences
- Plain‐language summaries for public dissemination

## 4 Common Pitfalls and How to Address Them: Updated RRMG Recommendations

Rapid reviews must balance speed with rigor. To maintain credibility and usability, it is critical to anticipate and address common challenges using structured guidance. The 2024 Cochrane RRMG update offers 24 core recommendations to guide this process, many of which are designed to prevent typical pitfalls (Table 3) [1].

**TABLE 3:** Common pitfalls and mitigation strategies.

Pitfall	Mitigation Strategy
Inadequate interest holder input	Engage interest holders early and at key decision points. Schedule feedback check‐ins during the review.
Potential for restricted scope to introduce bias	Describe and justify all limits (e.g., language, dates, outcomes) and discuss implications.
Methods not transparent	Follow reporting standards (e.g., PRISMA).
Poor team calibration	Pilot all screening and extraction forms; continuous training.
Methodological shortcuts without justification	Explain all restrictions (e.g., single screener, limited databases). Include rationale in the protocol and report.
Over‐reliance on automation	Use automation tools to support—but not replace—manual processes. Verify outputs.
Unassessed confidence in findings	Apply GRADE where feasible for key outcomes. If not, openly state limitations in evidence certainty.

By following Cochrane RRMG updated recommendations and mitigation strategies, review teams can maintain quality and transparency—ensuring timely reviews, maintaining rigor, and identifying possible biases introduced. The following practical checklist serves as a quick reference only (Table 4). To ensure methodological rigor, teams should rely on the full Cochrane rapid review methods guidance when implementing these recommendations [1].

**TABLE 4:** Quick‐reference checklist based on key recommendations from RRMG (2024).

Quick‐Reference Checklist for Rapid Reviews
Engage interest holders early and regularly
Define a narrow, focused PICO with a limited number of key outcomes
Draft protocol with clear, justified restrictions
Search ≥ 2 databases
Pilot and calibrate screening and data extraction
Use dual (or verified single) screening
Extract core data; assess risk of bias by one person, with verification by a second person
Apply GRADE (if feasible); explain confidence levels
Use collaborative tools and semi‐automation keeping humans in the loop
Synthesize (narrative or meta‐analysis)
Report transparently following reporting standards (e.g., PRISMA)
Log methods, decisions, restrictions, and changes

### 4.1 A Dynamic Process—Adapting as You Go

Rapid reviews may be adapted as they progress. Early findings from screening or searching may require adjustments to the protocol, scope, or methods. Maintaining a clear decision log and engaging interest holders throughout helps ensure transparency, accountability, and trust in the process. This underscores the dynamic and iterative nature of rapid reviews, in which methodological decisions may be refined in response to emerging insights.

## 5 Acknowledging Other Rapid Review Approaches

Although rapid methodologies can be applied across the full range of evidence synthesis types [19], this tutorial focuses specifically on rapid reviews of interventions (typically assessing effectiveness and safety using quantitative studies). It is important to recognize the growing use of a rapid approach for other types of evidence syntheses, such as rapid qualitative evidence syntheses [20] and rapid scoping (or “big picture”) reviews [21]. These approaches explore experiences, implementation, context, and evidence breadth. Although they share methodological shortcuts with intervention reviews, each has distinct goals and methods [19]. Clear reporting is essential to avoid confusion and ensure alignment between the review type and its intended decision‐making purpose.

## 6 Conclusion

Rapid reviews are increasingly important tools in health decision‐making, offering timely and relevant evidence to inform decision‐making. A well‐conducted rapid review transparently balances:

- Speed versus rigor
- Comprehensiveness versus feasibility
- User needs versus evidence limitations

While methodological shortcuts are inherent, their risks can be allayed through transparency, interest holder engagement, and adherence to best practices. The RRMG's evolving guidance and rapid reviews methods series in BMJ's Evidence‐Based Medicine provide a robust foundation for conducting rapid reviews that are both efficient and credible.

### 6.1 Further Reading and Online Content

*Module 13: Rapid reviews. In: Cochrane Interactive Learning: Conducting an intervention review. Cochrane, 2025*.

Available from: interactivelearning.cochrane.org/module-13.

This learning module introduces the key principles of rapid reviews, including how they differ from systematic reviews and when they are most appropriate. Participants will learn practical approaches for involving knowledge users, using methodological short‐cuts, applying supportive tools, and clearly communicating results. Access is available through a paid subscription to Cochrane's full online interactive learning content. However, it is free for active Cochrane Authors.

*Cochrane Live Learning Events – Rapid Reviews Collection*

Available from: www.cochrane.org/learn/webinars/collection/2917.

This collection of webinars showcases leading experts discussing practical and methodological aspects of rapid reviews, from scoping, qualitative synthesis, and literature searching to team processes, bias assessment, and stakeholder involvement. It also highlights applications in health policy, emergent decision‐making, and future directions for advancing rapid review methods.

*Devane D, Hamel C, Gartlehner G*, et al. *Key concepts in rapid reviews: an overview. J Clin Epidemiol. 2024;175:111518. doi: 10.1016/j.jclinepi.2024.111518*

This paper describes how rapid reviews are conducted, outlining their key steps, strengths, and limitations, and illustrates their use with a real‐world example. It also highlights the need for continued research to improve and standardize these methods.

## 7 Microlearning Module

To accompany the tutorial, please see the following URL for access to the microlearning module: ‐ https://share.gomolearning.com/sharelink/f107682b9ae69261fd4ad8ca80b3e6f19417241d145b86676f/.

## Author Contributions

**Chantelle Garritty:** conceptualization, methodology, writing – original draft, writing – review and editing, project administration. **Candyce Hamel:** conceptualization, methodology, writing – original draft, writing – review and editing. **Barbara Nussbaumer‐Streit:** conceptualization, methodology, writing – original draft, writing – review and editing.

## Funding

The authors have nothing to report.

## Conflicts of Interest

Chantelle Garritty, Candyce Hamel, and Barbara Nussbaumer‐Streit are Co‐Convenors of the Cochrane Rapid Reviews Methods Group. The authors have previously co‐authored several publications in *BMJ Evidence‐Based Medicine* (2024) as part of its rapid review methods series and contributed to the development of the updated Cochrane Rapid Reviews Methods Guidance, published in *The BMJ* in 2024. While these prior publications informed the conceptual foundations of this work, the content presented in this chapter is original and has been specifically developed for this tutorial. In addition, Barbara Nussbaumer‐Streit is Co‐Director of Cochrane Austria, and Candyce Hamel is an Associate Convenor of the Joint AI Methods Group. Chantelle Garritty and Candyce Hamel also serve as Methods Group representatives on the Cochrane Methods Executive Board. Chantelle Garritty is a consulting advisor with Cochrane Response and an Associate Editorial Board Member of *Cochrane Evidence Synthesis Methods*.

## Data Availability

Data sharing not applicable to this article as no datasets were generated or analyzed during the current study.
